# Comprehensive Curative Effect of Targeting PD-1 or Traditional Single-Agent Chemotherapy in Second-Line Therapy for Terminal or Metastatic Esophageal Cancer: A Systematic Review and Meta-Analysis

**DOI:** 10.1155/2022/4033863

**Published:** 2022-08-25

**Authors:** Yidan Wang, Qiuxing Yang, Jia Liu, Xiying Shen, Guomei Tai, Hongmei Gu

**Affiliations:** ^1^Department of Radiology, Affiliated Hospital of Nantong University, Nantong University, Nantong, China; ^2^Cancer Research Center Nantong, Nantong Tumor Hospital, Affiliated Tumor Hospital of Nantong University, Nantong, China; ^3^Department of Radiotherapy, Nantong Tumor Hospital, Affiliated Tumor Hospital of Nantong University, Nantong, China

## Abstract

The number of programmed cell death protein 1 (PD-1) inhibitors is gradually increasing; this study aimed to comprehensively and systematically evaluate the impact of PD-1 inhibitors as second-line therapy for terminal or metastatic esophageal cancer (EC) on patient survival and the occurrence of adverse events. Suitable randomized controlled trials (RCTs) were retrieved from PubMed, Web of Science, Embase, and Cochrane Library databases. Moreover, we searched for conference abstracts from the American Society of Clinical Oncology (ASCO) and the European Society for Medical Oncology (ESMO) to compare the comprehensive curative effects of PD-1 inhibitors or single-agent therapy in terminal or metastatic EC. The primary outcome indicators were overall survival (OS), progression-free survival (PFS), objective response rate (ORR), and disease control rate (DCR). Treatment-related adverse events (TRAEs) were the secondary outcome indicators. We compared the previously mentioned indicators of the two treatment modalities using Stata software (version 12.0). We compared the long-term survival rates of both treatment groups and analyzed the possible factors affecting OS. We selected five RCTs with 2197 patients as study subjects. Compared with conventional single-agent chemotherapy, PD-1 inhibitors greatly improved the patients' OS (HR = 0.77, 95% CI 0.70–0.85, *P* < 0.001), but PFS (HR = 0.93, 95% CI 0.77–1.12, *P*=0.431) and DCR (RR = 0.93, 95% CI 0.71–1.22, *P*=0.609) were not greatly improved. Moreover, PD-1 inhibitors improved ORR (RR = 1.83, 95% CI 1.16–2.89, *P*=0.009) and decreased TRAEs (RR = 0.76, 95% CI 0.61–0.95, *P* < 0.001) and serious TRAEs (RR = 0.40, 95% CI 0.32–0.49, *P* < 0.001). Further analysis demonstrated that OS was affected by age, sex, region, smoking history, and the number of organ and lymph node metastases. Compared with the traditional single chemotherapy drugs, PD-1 inhibitors can achieve higher OS and ORR, fewer and more serious TRAEs, and better efficacy and safety for second-line therapy of terminal or metastatic EC.

## 1. Introduction

According to the World Cancer Report 2020 released by the World Health Organization (WHO) in 2021, esophageal cancer (EC) ranks the eighth, and the mortality rate ranks the sixth. The histological subtype of 90% of EC cases in Asia and Africa is esophageal squamous cell carcinoma (ESCC), and the rest is esophageal adenocarcinoma (EAC) [1, 2]. ESCC patients' prognosis is poor, with a five-year overall survival rate of less than 15% [3]. Chemotherapy is often the conventional second-line therapy for terminal or metastatic EC [4]. Fluorouracil combined with platinum is the preferred therapy for locally terminal or metastatic EC [5, 6]. If first-line treatment is ineffective, the second-line therapy is usually monotherapy [5]. Commonly used second-line drugs include paclitaxel, docetaxel, and irinotecan [7–9]. However, these drugs can cause leukopenia, neutropenia, and neurotoxicity [10–12]. Therefore, there is an urgent need to identify second-line chemotherapy drugs that can improve the prognosis of terminal or metastatic EC.

In recent years, EC immunotherapy has attracted increasing attention [13]. By binding to protein receptors on the surface of T cells, immune checkpoint inhibitors (ICIs) activate immune checkpoints activated by T cells, restore the activity of T cells, and enhance the body's antitumor immune response [14, 15]. Programmed cell death protein 1 (PD-1), programmed death-ligand 1 (PD-L1), and cytotoxic T-lymphocyte antigen-4 (CTLA-4) are the most basic immune checkpoints in EC and can be inhibited by monoclonal antibodies, which can be used as targets for EC immunotherapy [16]. Some experiments have demonstrated that high expression of PD-1, PD-L1, and CTLA-4 is closely associated with a poor prognosis of EC [17–19]. Therefore, targeting the CTLA-4 or PD-1/PD-L1 pathway can be used to treat EC. PD-1 inhibitors are popular in clinical practice and have broad clinical applications. Since 2019, the U.S. Food and Drug Administration (FDA) has approved the PD-1 inhibitors pembrolizumab and nivolumab for EC treatment [20, 21]. Camrelizumab and sintilimab have also been studied as second-line therapy for ESCC [22, 23]. Although multiple clinical studies have shown that PD-1 inhibitors can effectively treat EC, PD-1 inhibitors still cause different drug-related adverse reactions [24].

With the wider use of PD-1 inhibitors in clinical practice, more attention has been paid to the comprehensive curative effects of PD-1 inhibitors. It is urgent to determine whether PD-1 inhibitors can effectively improve the efficacy of EC while producing minimal posttreatment adverse events. To evaluate the curative effect of PD-1 inhibitors in EC more comprehensively and systematically, this study selected randomized controlled trials (RCTs) of PD-1 inhibitors as second-line monotherapy drugs for EC. Efficacy and safety were evaluated in detail using systematic reviews and meta-analyses, aiming to provide reliable and necessary evidence-based medicine for second-line therapy of EC.

## 2. Materials and Methods

### 2.1. Search Strategy

A comprehensive search of the published literature was conducted using PubMed, Web of Science, Embase, and Cochrane Library databases. In addition, conference abstracts from the American Society of Clinical Oncology (ASCO) and the European Society for Medical Oncology (ESMO) were searched. All RCTs of EC patients treated with PD-1 inhibitors and conventional second-line chemotherapeutics between January 2015 and April 2022 were collected. The search language used was English. The following retrieval strategies were used: esophageal neoplasms, esophageal cancer, immune checkpoint inhibitors, immune checkpoint blockade, programmed cell death 1 receptor, randomized controlled trials, and PD-1 inhibitors. The protocol of this systematic review and meta-analysis was registered on the INPLASY platform (registration number: INPLASY202270019). The search strategies in the Web of Science are shown in Supplementary Table S1.

### 2.2. Inclusion and Exclusion Criteria

Qualified literature was included in this study according to the following criteria: (1) RCTs of locally terminal or metastatic EC that became more severe after first-line therapy; (2) the PD-1 inhibitor treatment group used a single PD-1 inhibitor for chemotherapy, and the chemotherapy group used conventional EC second-line chemotherapy drugs; (3) trials included at least three of the following outcomes: overall survival (OS), progression-free survival (PFS), objective response rate (ORR), disease control rate (DCR), and treatment-related adverse events (TRAEs). Reviews, repeated studies, case reports, non-RCTs, animal studies, irrelevant studies, and studies for which no valid data could be obtained were excluded. If there was an overlap in the data, we only chose the one with the most complete data. Two investigators selected the relevant articles based on the inclusion and exclusion criteria.

### 2.3. Data Fetch and Quality Evaluation

Data fetch and quality evaluation were independently obtained and evaluated from each study by two authors, and disagreements were resolved through discussion among all group members. The following information was extracted from each included study: first author, trial name, trial stage, treatment, number of patients, age, sex, region, OS, PFS, ORR, DCR, TRAEs, Eastern Cooperative Oncology Group performance status (ECOG PS), histological type, organ metastasis, lymph node metastasis, and PD-L1 expression. In addition, we conducted a risk assessment of RCTs using the Cochrane Risk of Bias Assessment Tool [25].

### 2.4. Statistical Analyses

All data were statistically analyzed using Stata 12.0 and Review Manager 5.3. All OS and PFS used HRs and 95% confidence intervals (CIs) as the effect size of statistical indicators. RRs and 95% CIs were used for other variables, such as ORR, DCR, and TRAEs. The heterogeneity assessment of these studies was performed using Cochran's *Q* test or Higgins *I*^2^ statistic. If *P* < 0.1 or *I*^2^ > 50%, it was taken for high heterogeneity among the studies, and the analysis would use the random-effects model; otherwise, the fixed-effects model would be used. When heterogeneity was observed between studies, subgroup analyses were performed to identify the sources of heterogeneity. When *P* < 0.05, the differences were considered statistically significant. After excluding each study individually, the combined effect size was reestimated and compared with the results of the meta-analysis before the exclusion, and the impact of the study on the combined effect size and the robustness of the meta-analysis results were discussed. Publication bias was detected using Egger's and Begger's tests. If *P* > 0.05, publication bias did not exist; otherwise, there was a publication bias.

## 3. Results

### 3.1. Search Results and Quality Evaluation

**Figure 1:**
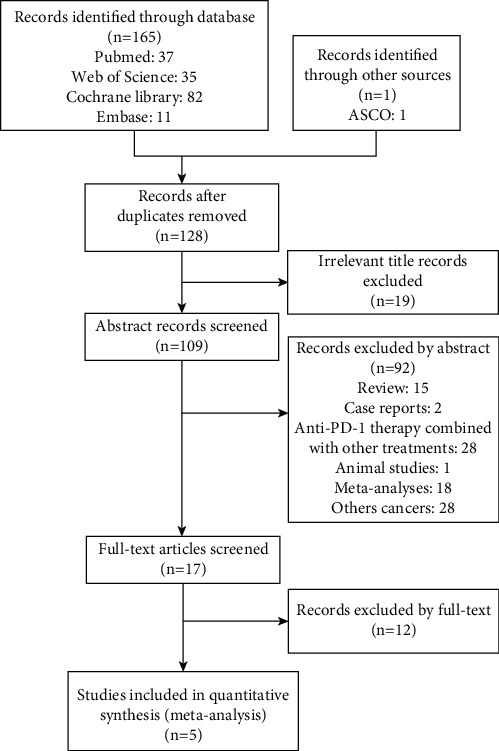
The flowchart of the retrieval process.

**Figure 2:**
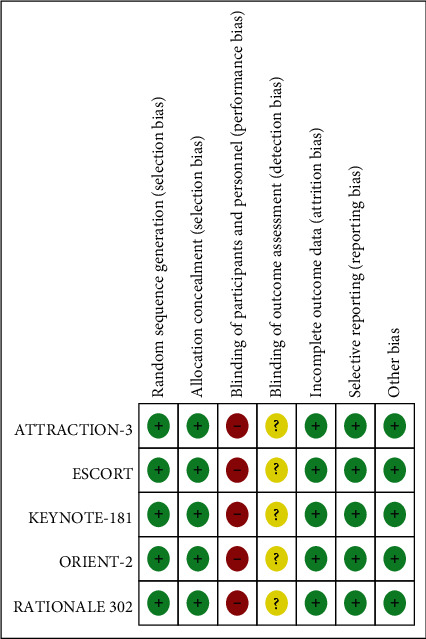
Quality evaluation of all included articles using revman 5.3.

We retrieved 166 papers from major databases and conferences. After preliminary screening, 38 duplicate studies were excluded, and 111 studies were excluded after reading the general information. Then, we read the full text of the remaining 17 papers and finally included five RCTs that met the requirements.  Figure 1 shows the flowchart of the retrieval process. The quality of the included studies was also assessed. As shown in Figure 2, the quality assessment of all the included studies was relatively low.

### 3.2. Basic Characteristics of the Selected Studies

These five studies were all prospective RCTs, among which ORIENT-2 was a phase 2 clinical trial, and the rest were phase 3 clinical trials. ESCORT and ORIENT-2 were conducted in China, and the other three were carried out worldwide. The basic characteristics of the selected studies are summarized in Table 1, and the survival outcomes in each study are presented in Table 2.

### 3.3. Primary Outcome Measures

**Figure 3:**
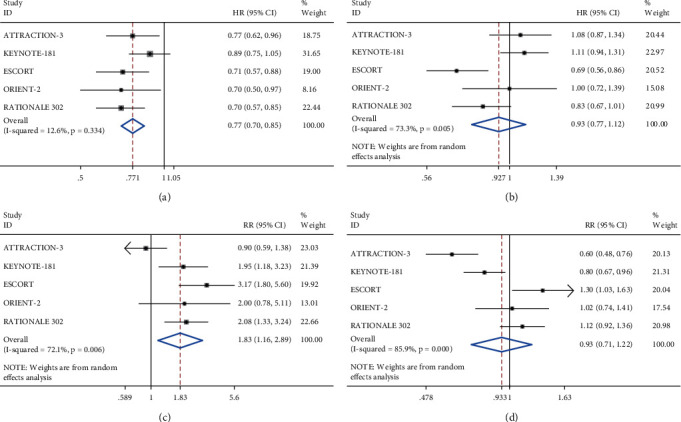
Meta-analysis results of the primary outcome measure with PD-1 inhibitors and with monotherapy. (a) Overall survival (OS); (b) progression-free survival (PFS); (c) objective response rate (ORR); (d) disease control rate (DCR).

The results of several studies showed that PD-1 inhibitors were more beneficial in improving the OS of patients than traditional chemotherapy drugs (HR = 0.77, 95% CI 0.70–0.85, *P* < 0.001) (Figure 3(a)). The combined PFS results showed that the heterogeneity among the studies was relatively large (*I*^2^ = 73.3%, *P*=0.005) (Figure 3(b)); therefore, we used a random-effects model for meta-analysis. The results showed that there was no obvious statistical difference between the immunotherapy group and the single-agent chemotherapy group (HR = 0.93, 95% CI 0.77–1.12, *P*=0.431), suggesting that PD-1 inhibitors had no significant effect on improving PFS. The heterogeneity test of ORR and DCR data both showed large heterogeneity; therefore, we used a random-effects model. In contrast, treatment with PD-1 inhibitors could achieve a higher ORR (RR = 1.83, 95% CI 1.16–2.89, *P*=0.009), whereas DCR was not significantly different (RR = 0.93, 95% CI 0.71–1.22, *P*=0.609) (Figures 3(c) and 3(d)).

### 3.4. Long-Term Survival Comparison

**Figure 4:**
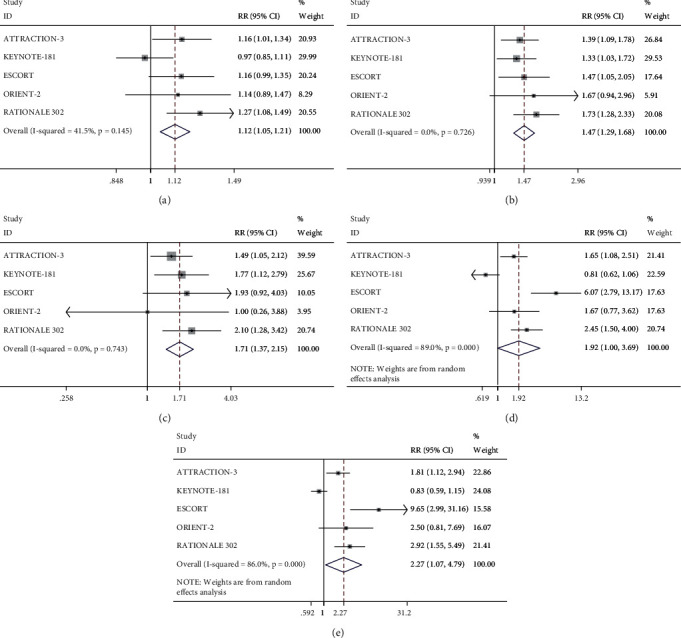
Meta-analysis results of OS rate and PFS rate with PD-1 inhibitors and with monotherapy at different times. (a) The six-month OS rate; (b) the 12-month OS rate; (c) the 18-month OS rate; (d) the six-month PFS rate; (e) the eight-month PFS rate.

A long-term survival analysis using the two drug treatments separately is shown in Figure 4. The six-month OS rate (RR = 1.12, 95% CI 1.05–1.21, *P*=0.001), 12-month OS rate (RR = 1.47, 95% CI 1.29–1.68, *P* < 0.001), and 18-month OS rates (RR = 1.71, 95% CI 1.37–2.15, *P* < 0.001) were higher with PD-1 inhibitors than with monotherapy. Analysis of PFS data showed that the six-month PFS rate (RR = 1.92, 95% CI 1.00–3.69, *P*=0.050) was not affected by PD-1 inhibitors, but PD-1 inhibitors could improve the eight-month PFS rate (RR = 2.27, 95% CI 1.07–4.79, *P*=0.032).

### 3.5. Analysis of Influencing Factors of OS

To better investigate the factors affecting OS, we combined the data from the two treatment regimens and performed a subgroup analysis of nine factors that might affect OS (Table 3). According to the results of the subgroup analysis, age, sex, region, smoking history, PD-L1 expression, ECOG PS, organ metastasis, and lymph node metastasis influenced OS. However, there was a greater heterogeneity within the PD-L1 expression group and the ECOG PS group. At the same time, we found that PD-1 inhibitors tended to help improve OS.

### 3.6. Treatment-Related Adverse Events

**Figure 5:**
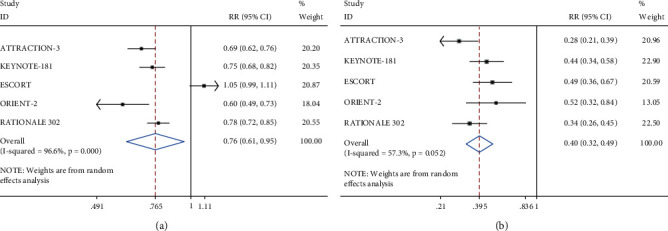
Meta-analysis of TRAEs with PD-1 inhibitors and with monotherapy. (a) Any grade of TRAEs; (b) grades 3–5 of TRAEs.

We compared TRAEs between PD-1 inhibitors and monotherapy. PD-1 inhibitors were associated with fewer TRAEs (RR = 0.76, 95% CI 0.61–0.95, *P* < 0.001) and grade 3–5 TRAEs (RR = 0.40, 95% CI 0.32–0.49, *P* < 0.001) (Figure 5). Common events that occurred in both groups were diarrhea, decreased appetite, nausea, anemia, and decreased white blood cell and neutrophil counts. The results of the combined five studies confirmed that immunotherapy significantly reduced the occurrence of these adverse events (Table 4).

### 3.7. Sensitivity Analysis and Publication Bias

**Figure 6:**
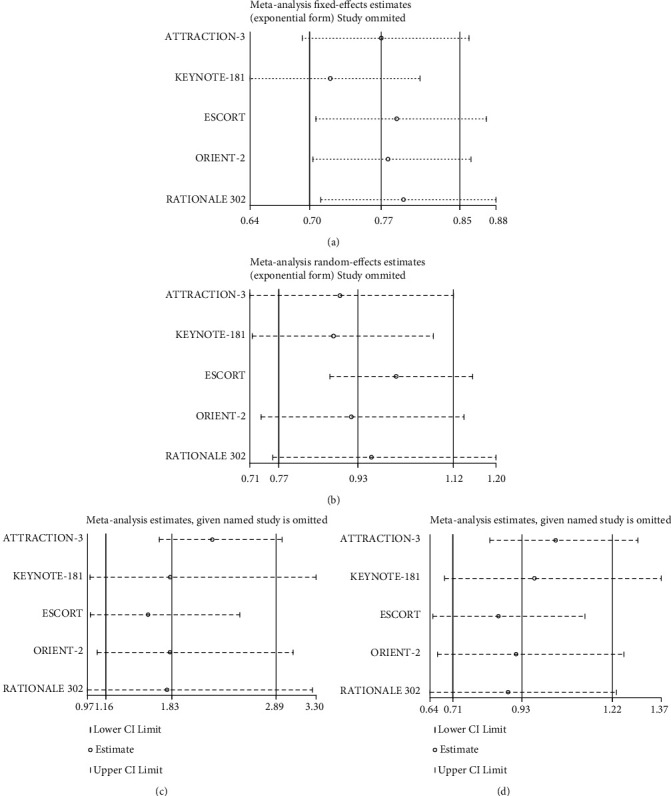
Sensitivity analysis of primary outcome measure. (a) OS; (b) PFS; (c) ORR; (d) DCR.

To evaluate the robustness of our findings, we conducted a sensitivity analysis of the primary outcome measures.  Figure 6 shows the results of the sensitivity analysis. The combined results for OS, PFS, ORR, and DCR were stable, indicating that the results of this study are stable and credible. Publication bias could not be assessed because few studies were included.

## 4. Discussion

With the gradual advancement of current molecular research on EC, increasing attention has been paid to EC immunotherapy [29]. PD-1 is present in memory CD8^+^ T cells, memory CD4^+^ T cells, mucosal-associated invariant T (MAIT) T-cells, gamma delta (Gd) T-cells, regulatory T-cells (Tregs), monocytes, and natural killer (NK) cells, which is a transmembrane protein [30, 31]. When PD-1 binds to its ligand, PD-L1, the two recruits, SHP2, generate inhibitory signals and inhibit the PI3K/AKT/mTOR and RAS/MEK/ERK1/2 pathways, thereby inhibiting the activation of T cells and helping the immune escape of cancer cells [32, 33]. In addition, PD-L1 is abundant in cancer cells, and various cytokines in the tumor microenvironment (TME) can trigger the production of PD-L1 [34]. PD-L1 in cancer cells can activate PI3K-AKT and MAPK signaling to promote EMT [35, 36]. Therefore, PD-1/PD-L1 inhibitors can interrupt immune escape and reactivate T cells for their anticancer activity.

Targeting the PD-1/PD-L1 pathway has become a sought-after treatment in tumor immunotherapy. PD-1 inhibitors currently used in ESCC include nivolumab, pembrolizumab, toripalimab, camrelizumab, sintilimab, and tislelizumab, and PD-L1 inhibitors include duvalumab and adebrelimab. According to some studies, most PD-1 inhibitor therapies can significantly prolong the OS of patients, and pembrolizumab can reduce drug-related adverse reactions. Research on toripalimab continues [37]. Regarding PD-L1 inhibitors, durvalumab and tremelimumab (a CTLA-4 inhibitor) combined with concurrent chemoradiotherapy (CCRT) are effective in patients with locally terminal ESCC [38], while adebrelimab combined with chemotherapy has shown good efficacy and safety in the first-line treatment of patients with locally advanced or metastatic ESCC [39]. Treatment with PD-1/PD-L1 inhibitors has a good effect on EC. In the future, tumor immunotherapy could be considered the first-choice treatment for EC.

In this study, we included five open-label RCTs using meta-analysis methods, selected PD-1 inhibitors, and single traditional chemotherapy drugs as second-line chemotherapy drugs for EC. The efficacy and safety after treatment were compared, and the final survival of the patients and the occurrence of adverse reactions were analyzed. Our findings suggest that PD-1 inhibitors achieve higher OS and ORR than single agents, suggesting that PD-1 inhibitors can prolong patient survival and effectively reduce tumor size. However, no obvious distinction was observed between PFS and DCR in traditional medicine. These five studies all used OS as the primary outcome measure, and the results of the KEYNOTE-181 study showed that immunotherapy did not have a better OS than traditional chemotherapy. Therefore, we conducted further analysis of OS to clarify the specific reasons for the difference in OS. We found that age, sex, region, smoking history, and the number of organs and lymph node metastases influenced OS, but OS was not related to histological type.

This may be because the number of EAC samples was too small, and the sample size could be increased in future studies. At the same time, we can see that, under the stratification of these factors, PD-1 inhibitors help improve OS compared with traditional chemotherapy. Among these factors, PD-L1 expression and ECOG PS were more heterogeneous. The difference in the expression of PD-L1 is due to the different detection methods of PD-L1 used in different studies, resulting in differences in the staining of PD-L1, which in turn affects the subjective judgment of the evaluators. Moreover, the criteria selected to evaluate PD-L1 expression in different studies are different, also affecting the intragroup heterogeneity of PD-L1 expression. The intragroup heterogeneity of the ECOG PS scores may be due to errors between reviewers in different studies.

We compared the long-term survival. Immunotherapy significantly improved OS rates at six, 12, and 18 months. Regarding PFS, the six-month PFS rate of the immunotherapy group was not significantly different from that of the traditional treatment group (*P*=0.05), but the eight-month PFS rate was obviously better than that of the traditional treatment group (*P*=0.032). This difference may be because PFS refers to the time from the start of a randomized clinical trial to any aspect of tumor progression or death from any cause, a process that requires long-term follow-up. The shorter the follow-up period, the greater the likelihood of bias. This was also demonstrated by the significant improvement in the eight-month PFS rate compared with the six-month PFS rate. Therefore, an extended follow-up time more accurately reflects the true effects of both treatments. In a meta-analysis of PD-1/PD-L1 inhibitors as monotherapy or combined with chemotherapy in advanced non-small cell lung cancer (NSCLC), combinations of PD-1/PD-L1 inhibitors and chemotherapy improved PFS and ORR compared with monotherapy [40]. This inspired us to combine PD-1 inhibitors with traditional chemotherapy for the treatment of terminal or metastatic EC. RCTs of PD-1 inhibitors combined with chemotherapy have been conducted in clinical settings [41, 42]. Existing research shows that combined chemotherapy can improve the OS and PFS of patients. However, in the KEYNOTE-590 study [42], combination chemotherapy resulted in more serious TRAEs.

The emergence of PD-1 inhibitors has improved the OS rate of the population and revolutionized cancer treatment. However, these drugs also have certain disadvantages. The most common TRAEs were diarrhea, reduced appetite, nausea, anemia, decreased white blood cell and neutrophil counts, and hypothyroidism. In the analysis of TRAEs, we found that PD-1 inhibitors significantly reduced the number of adverse events and serious adverse events. This finding is consistent with the results of several clinical trials that have been completed. The number of all-grade TRAEs caused by ICIs is less than that of traditional treatments, and their safety is relatively high [43, 44]. PD-1 inhibitors also have disadvantages, such as immune-related adverse events (IRAEs) and poor tumor tissue penetration [45]. As we know from the included studies KEYNOTE-181 and ORIENT-2 [23, 27], PD-1 inhibitor therapy resulted in more IRAEs.

IRAEs occur because PD-1 inhibitors can activate the immune system by activating T cells to attack cancer cells, but they can also attack normal cells. Therefore, in the future, we can develop small-molecule drugs targeting PD-L1, which can be combined with antibodies targeting other immune checkpoints to produce synergistic anticancer activity [46]. Alternatively, drugs can upregulate the expression of PD-L1 to enhance the efficacy of anti-PD-1/PD-L1 antibodies.

At the same time, we can also consider another hot field in combination tumor therapy, targeted therapy, which combines immunotherapy with targeted therapy. Low-dose VEGF inhibitors reduce the sprouting of immature blood vessels to normalize their structure and function, facilitate the delivery of chemotherapeutic drugs, and promote the penetration of killer T cells into tumors [47]. In the process of tumor angiogenesis, antiangiogenic drugs can reduce the generation of immunosuppressive factors and prevent the dysfunction of endothelial cells, thus improving the efficacy of immunotherapy [48]. Real-world clinical studies of antiangiogenic drugs in combination with PD-1 inhibitors for ESCC therapy [49]. This suggests that combination therapy with PD-1 inhibitors and antiangiogenic drugs is a promising strategy; however, due to the lack of more data, its efficacy, safety, and mechanism need to be further analyzed.

This study was a preliminary meta-analysis evaluating the comprehensive curative effect of PD-1 inhibitors, and the included studies were randomized open-label multicenter trials. We analyzed the primary outcome measures, as well as TRAEs, and concluded that PD-1 inhibitors are helpful in the treatment of terminal or metastatic EC. However, this study has some shortcomings. First, the number of included studies was insufficient. Second, most of the patients included in the study had ESCC, and there were fewer patients with EAC, which was not conducive to our judgment of the efficacy of different histological types. Finally, we could not assess the patients' health-related quality of life due to a lack of data. We look forward to more comprehensive RCTs to advance research on EC in the future.

## 5. Conclusions

Our findings suggest that PD-1 inhibitors significantly prolong OS in patients with terminal or metastatic EC as a second-line therapy, with more individuals achieving objective responses. Regarding TRAEs, treatment with PD-1 inhibitors occurred less frequently. Based on the previously mentioned results, we believe that PD-1 inhibitors as second-line chemotherapy drugs have better efficacy and safety than traditional drugs in treating terminal or metastatic EC; however, more clinical studies are necessary to support this view.

## Figures and Tables

**Table 1:** Basic characteristics of the selected studies.

RCTs	Trial code	Area	Phase	Participant	Group	No. of patients	Treatment regimen
ATTRACTION-3 [26]	NCT02569242	Global	III	ESCC	Immunotherapy chemotherapy	210 209	Nivolumab 240 mg, Q2W paclitaxel or docetaxel
KEYNOTE-181 [27]	NCT02564263	Global	III	ESCA	Immunotherapy chemotherapy	198 203	Pembrolizumab 200 mg, Q3W paclitaxel or docetaxel or irinotecan
ESCORT [22]	NCT03099382	China	III	ESCC	Immunotherapy chemotherapy	228 220	Camrelizumab 200 mg, Q2W docetaxel or irinotecan
ORIENT-2 [23]	NCT03116152	China	II	ESCC	Immunotherapy chemotherapy	95 95	Sintilimab 200 mg, Q3W paclitaxel or irinotecan
RATIONALE 302 [28]	NCT03430843	Global	III	ESCC	Immunotherapy chemotherapy	256 256	Tislelizumab 200 mg, Q3W paclitaxel or docetaxel or irinotecan

**Table 2:** Specific outcomes of survival measures for the selected studies.

RCTs	Groups	Median follow-up duration (months)	Median OS (months) (95% CI)	Median PFS (months) (95% CI)	ORR (%)	DCR (%)
ATTRACTION-3	Immunotherapy group chemotherapy group	10.5 8.0	10.9 (9.2–13.3) 8.4 (7.2–9.9)	1.7 (1.5–2.7) 3.4 (3.0–4.2)	19.3 21.5	37.4 62.7
KEYNOTE-181	Immunotherapy group chemotherapy group	7.1 6.9	7.1 (6.2–8.1) 7.1 (6.3–8.0)	2.1 (2.1–2.2) 3.4 (2.8–3.9)	13.1 6.7	38.5 48.1
ESCORT	Immunotherapy group chemotherapy group	8.3 6.2	8.3 (6.8–9.7) 6.2 (5.6–6.7)	1.9 (1.9–2.4) 1.9 (1.9–2.1)	20.2 6.4	44.7 34.5
ORIENT-2	Immunotherapy group chemotherapy group	7.2 6.2	7.2 (5.8–9.7) 6.2 (5.4–7.9)	1.6 (1.5–2.8) 2.9 (2.6–3.6)	12.6 6.3	44.2 43.2
RATIONALE 302	Immunotherapy group chemotherapy group	8.5 5.8	8.6 (7.5–10.4) 6.3 (5.3–7.0)	1.6 (1.4–2.7) 2.1 (1.5–2.7)	20.3 9.8	46.9 41.8

**Table 3:** Analysis of factors affecting OS in PD-1 inhibitor group and chemotherapy group.

Factors	No. of studies	No. of patients	HR	95% CI	*I* ^2^ (%)	*P* value
Age, year			0.77	0.70–0.84	0	<0.001
<65	5	1357	0.75	0.67–0.85		
≥65	5	840	0.79	0.68–0.92		
Sex			0.76	0.64–0.88	35.5	<0.001
Male	5	1912	0.79	0.72–0.87		
Female	5	285	0.66	0.50–0.87		
Region			0.73	0.64–0.83	0	<0.001
Asia	3	1048	0.73	0.64–0.83		
Ex-Asia	3	511	0.74	0.43–1.29		
History of smoking			0.70	0.59–0.82	0	<0.001
Never	3	257	0.75	0.56–0.99		
Former/current	3	863	0.67	0.55–0.82		
Histology			0.89	0.58–1.35	88.1	0.571
Squamous cell carcinoma	5	1970	0.73	0.66–0.81		
Adenocarcinoma	1	227	1.12	0.85–1.47		
PD-L1 expression			0.74	0.57–0.97	83.3	0.028
Low	5	1409	0.84	0.75–0.95		
High	5	621	0.64	0.54–0.77		
ECOG PS			0.75	0.61–0.92	73.4	0.007
0	5	713	0.84	0.71–1.00		
1	5	1482	0.68	0.60–0.78		
Number of organs with metastases			0.73	0.61–0.86	18	<0.001
1	2	359	0.81	0.63–1.03		
≥2	2	508	0.68	0.56–0.82		
Lymph node metastasis			0.74	0.64–0.87	0	<0.001
No	2	405	0.68	0.55–0.86		
Yes	2	462	0.80	0.65–0.98		

**Table 4:** Combined results of common adverse events with PD-1 inhibitors and with monotherapy.

TRAEs	RR	95% CI	*P* value
Diarrhea	0.28	0.14–0.56	<0.001
Decreased appetite ACT	0.24	0.13–0.43	<0.001
Nausea	0.12	0.05–0.26	<0.001
Anemia	0.21	0.14–0.32	<0.001
White blood cell count decreased	0.07	0.03–0.17	<0.001
Neutrophil count decreased	0.07	0.03–0.14	<0.001

## Data Availability

All the data generated or analyzed during this study are included within the article.

## Supplementary Materials

Supplementary Materials Supplementary Table S1: search strategies in the web of science.
