# Supplementary material for: Comprehensive Curative Effect of Targeting PD-1 or Traditional Single-Agent Chemotherapy in Second-Line Therapy for Terminal or Metastatic Esophageal Cancer: A Systematic Review and Meta-Analysis
Source: J Oncol. 2022 Aug 25;2022:4033863. doi: 10.1155/2022/4033863 (PMC9436578; doi:10.1155/2022/4033863)
Supplement: Supplementary Materials — Supplementary Table S1: search strategies in the web of science. [file 4033863.f1.docx]

**Supplementary File**

**Comprehensive curative effect of targeting PD-1 or traditional single-agent chemotherapy in second-line therapy for terminal or metastatic esophageal cancer：A systematic review and meta-analysis**

Supplementary Table S1. Search strategy

| **Database** | **Step** | **Search strategy** |
| --- | --- | --- |
| Web of Science | #1 | TS=(Esophageal Neoplasms OR Esophageal Neoplasm OR Neoplasm, Esophageal OR Esophagus Neoplasm OR Esophagus Neoplasms OR Neoplasm, Esophagus OR Neoplasms, Esophagus OR Neoplasms, Esophageal OR Cancer of Esophagus OR Cancer of the Esophagus OR Esophagus Cancer OR Cancer, Esophagus OR Cancers, Esophagus OR Esophagus Cancers OR Esophageal Cancer OR Cancer, Esophageal OR Cancers, Esophageal OR Esophageal Cancers) |
|  | #2 | TS=(Immune Checkpoint Inhibitors OR Checkpoint Inhibitors, Immune OR Immune Checkpoint Inhibitor OR Checkpoint Inhibitor, Immune OR Immune Checkpoint Blockers OR Checkpoint Blockers, Immune OR Immune Checkpoint Blockade OR Checkpoint Blockade, Immune OR Immune Checkpoint Inhibition OR Checkpoint Inhibition, Immune OR PD-L1 Inhibitors OR PD L1 Inhibitors OR PD-L1 Inhibitor OR PD L1 Inhibitor OR Programmed Death-Ligand 1 Inhibitors OR Programmed Death Ligand 1 Inhibitors OR PD-1-PD-L1 Blockade OR Blockade, PD-1-PD-L1 OR PD 1 PD L1 Blockade OR CTLA-4 Inhibitors OR CTLA 4 Inhibitors OR CTLA-4 Inhibitor OR CTLA 4 Inhibitor OR Cytotoxic T-Lymphocyte-Associated Protein 4 Inhibitors OR Cytotoxic T Lymphocyte Associated Protein 4 Inhibitors OR Cytotoxic T-Lymphocyte-Associated Protein 4 Inhibitor OR Cytotoxic T Lymphocyte Associated Protein 4 Inhibitor OR PD-1 Inhibitors OR PD 1 Inhibitors OR PD-1 Inhibitor OR Inhibitor, PD-1 OR PD 1 Inhibitor OR Programmed Cell Death Protein 1 Inhibitor OR Programmed Cell Death Protein 1 Inhibitors) |
|  | #3 | TS=(Programmed Cell Death 1 Receptor OR PD-1 Protein OR PD 1 Protein OR PD-1 Receptor OR PD 1 Receptor OR Receptor, PD-1 OR Antigens, CD279 OR CD279 Antigens OR CD279 Antigen OR Antigen, CD279 OR PD1 Receptor OR Receptor, PD1 OR Programmed Cell Death Protein 1 OR Programmed Cell Death 1 Protein) |
|  | #4 | TS=(randomized controlled trial OR randomized OR placebo) |
|  | #5 | #1 AND #2 AND #3 AND #4 |

* We systematically searched for the relevant literatures published before 18 April 2022 in the above database.
